# Rapid Oxidation of Adsorbed Organic Impurities on Stainless Steel by Treatment with Diluted Peroxynitric Acid

**DOI:** 10.3390/ma18214984

**Published:** 2025-10-31

**Authors:** Jernej Ekar, Miran Mozetič, Janez Kovač, Nina Recek, Satoshi Ikawa, Katsuhisa Kitano

**Affiliations:** 1 Jozef Stefan Institute, Jamova Cesta 39, SI-1000 Ljubljana, Slovenia; miran.mozetic@ijs.si (M.M.); janez.kovac@ijs.si (J.K.); nina.recek@ijs.si (N.R.); 2 Osaka Research Institute of Industrial Science and Technology, 2-7-1 Ayumino, Izumi, Osaka 594-1157, Japan; ikawa@orist.jp; 3 Graduate School of Engineering, Osaka University, 2-1 Yamada Oka, Suita, Osaka 565-0871, Japan; kitano@ppl.eng.osaka-u.ac.jp

**Keywords:** peroxynitric acid, stainless steel cleaning, passivation, oxidation, SIMS, AFM, surface modifications

## Abstract

Stainless steel forms a native film of mixed metal oxides, and organic impurities are likely to adsorb on the surface upon exposure to ambient conditions. For many applications, oxides and impurities should be removed, and several techniques have been used for decades. An innovative method is presented in this paper. The organic impurities were oxidized using a water solution of 1 M peroxynitric acid (PNA). Stainless steel samples were immersed in the solution, and the oxidation of organic impurities was evaluated by the ultra-thin depth profiling using secondary ion mass spectrometry (SIMS). A minute of treatment with PNA caused oxidation of organic impurities and a decrease in the SIMS CN^–^ signal over an order of magnitude. Prolonged treatment caused the selective removal of the native iron oxide film, leaving a protective film of chromium oxide. Removal of the iron oxide film was also observed when stainless steel was treated with 1 M HNO_3_. The PNA method is useful for routine cleaning of stainless steel to remove the organic contaminants from the surface and keep the passive chromium oxide film intact. It is ecologically friendly and enables rapid decomposition of the traces of organic impurities likely to be adsorbed on the metallic surfaces.

## 1. Introduction

The surfaces of samples made from metals, alloys, and many other compounds are likely to be contaminated with a very thin film of organic impurities [1]. Stainless steel will also form a native oxide film [2,3]. The oxide film is rich in both chromium and iron oxides and often also in oxides of other elements in the stainless steel [4]. The elemental distribution on the nanoscale may influence the material properties [5]. The impurities could be removed effectively by treatment with energetic ions, which cause reasonably non-selective removal of the entire surface film. The ion-beam treatment is a standard method for etching solid materials by sputtering and is usually used for depth profiling for the determination of the thin film composition by advanced surface-sensitive techniques such as Auger-electron spectroscopy (AES), X-ray photoelectron spectrometry (XPS), and secondary ion mass spectrometry (SIMS) [6,7,8]. The surface of the sputtered samples remains free from impurities as long as ultra-high vacuum conditions are maintained, but even a short exposure to the ambient atmosphere will cause the formation of the native oxide film and adsorption of organic vapors, which are always present in ambient air. The thickness of the organic impurity layer is often about a monolayer since the chemical nature facilitates the irreversible adsorption of molecules on the metallic or oxidized metallic surfaces [9].

Numerous methods for cleaning stainless steel samples have been invented, and many are used in laboratory as well as industrial practice [10,11]. One of the novel techniques is plasma cleaning [12], while the most common method for degreasing (removal of organic impurities) is the application of non-polar liquids. A thin layer of inorganic impurities, like oxides, will likely be removed by etching in concentrated acids. When highly concentrated, the acids are likely to cause etching, but many low-molar acid solutions are not known as significant etchers [13,14]. Oxide layers can be effectively removed with the application of laser cleaning as well [15].

Nitric acid is sometimes used as a mild etcher. High-grade stainless steels are not affected much by diluted nitric acid. The poor etching is due to the passive layer of oxides formed upon stainless steel exposure to diluted nitric acid. The passive layer is often a few nm thick and contains various metal oxides. The most common are chromium and iron oxides. The breakdown of the passive oxide film will cause bulk corrosion but is unlikely to occur when the high-grade stainless steel is exposed to nitric acid at a concentration below 10 M at room temperature [16].

The treatment with nitric acid will not remove the adsorbed organic impurities since the acid alone does not interact chemically with most organic materials. Washing in detergents is a standard method for rough degreasing of products made from stainless steel. Traces of organic impurities are usually removed by the application of several baths containing non-polar solvents since the cleanliness of the surface is crucial for further modifications, especially in relation to adhesion [17]. The surface finish of products treated by standard methods is adequate for numerous applications, such as painting, printing, gluing, and the like. Still, the stainless steel surface is not free from organic impurities. Numerous authors reported traces of such impurities when examining metals with surface-sensitive techniques like AES, XPS, and SIMS [18].

In this paper, we present an alternative technique for the removal of both the layer of organic impurities and metal oxides other than chromium oxide. The samples were treated in a water solution of 1 M peroxynitric and nitric acids. This concentration is obtained using the standard procedure for synthesizing peroxynitric acid (PNA) on the laboratory scale. The higher the concentration, the more effective the PNA treatment, and the synthesis approach applied cannot provide concentrations above 1 M. The PNA, which is one of the reactive oxygen and nitrogen species (RONS), enabled fast oxidation and, thus, removal of the organic film, and the nitric acid caused a preferential removal of the iron oxide film.

## 2. Materials and Methods

Commercially available stainless steel foil with a thickness of about 100 µm was cut into rectangular pieces of dimension 10 × 10 mm^2^ and cleaned with a mixture of analytically pure acetone and isopropanol. Steel samples were initially wiped with the paper cloth soaked with the two solvents. Then they were submerged in the acetone and isopropanol mixture for approximately 10 min at room temperature and periodically mixed. Finally, they were once more rinsed with pure acetone and left to dry under ambient conditions. Samples made out of two different types of stainless steel were analyzed to confirm the wider applicability of the PNA treatment. The samples were rather smooth, as revealed by atomic force microscopy (AFM). We used an AFM microscope, Solver PRO (NT-MDT, Moscow, Russia), in a semi-contact mode. The surface morphology was measured over areas of 2 × 2 and 10 × 10 µm^2^, and the surface roughness *S*_a_ was calculated after subtraction of the corresponding plane.

### 2.1. PNA Synthesis and Treatment

The 1 M PNA solution was prepared as follows: First, a mixture of 22 mL of 30% hydrogen peroxide, 17 mL of 4 M nitric acid, and 14 mL of distilled water was cooled down to a temperature of approximately −10 °C using a mixture of ethanol and isopropanol at −80 °C as a cooling agent. Then, 13 mL of 40% NaNO_2_ solution was slowly added to the mixture of H_2_O_2_, HNO_3_, and distilled water. The addition of NaNO_2_ was performed gradually at the rate of 1 mL/min and during an intense mixing of the reaction mixture. NaNO_2_ has to be added slowly during mixing and cooling to prevent degradation of the synthesized PNA and to ensure its high concentration in the reaction mixture, which contains PNA, NaNO_3_, an excess of unreacted H_2_O_2_, and small amounts of HNO_3_ after the completion of the synthesis. The temperature during the synthesis was consequently kept between −10 and 0 °C. The pH was kept at approximately 0 since acidity is another factor contributing to the PNA stability. Namely, the PNA tends to decompose spontaneously at elevated pH and/or temperature. The detailed synthesis procedure is further explained elsewhere [19].

The concentration of PNA was measured with the colorimetric DPD assay, which utilizes the oxidation of the *N*,*N*-diethyl-*p*-phenylenediamine (DPD) in the presence of PNA. The resulting reaction mixture is colored, and the absorbance is measured at 553 nm. The DPD assay requires extensive dilution of the PNA reaction mixture to obtain an adequate absorbance range. In the first step, 100 µL of PNA mixture is mixed with 900 µL of distilled water cooled to 0 °C. In the second step, 15 µL of the solution prepared via the first step is mixed with 985 µL of distilled water cooled to 0 °C. In the last step, 20 µL of the solution prepared in the first two steps is added to 280 µL of the DPD assay mixture at room temperature and immediately mixed. This leads to the overall dilution ratio of 10,000. DPD assay mixture is prepared by mixing 20 µL of DPD solution with a concentration of 50 g/L, 30 µL of 200 mM sodium citrate buffer with a pH of 5, and 230 µL of distilled water. Determination of the absolute concentration of PNA can be accomplished by specially developed ion chromatography, and the results of the colorimetric DPD assay can be converted to the absolute concentration. Further details of the DPD assay are explained elsewhere [19].

The samples were treated with an as-prepared PNA reaction mixture, that is, a water solution of 1 M PNA and 1 M NaNO_3_, containing some nitric acid and H_2_O_2_. Rectangular steel foil pieces were dropped into the concentrated PNA solution at slightly above 0 °C. Cooling of the mixture was stopped after the addition of the samples, and only intense mixing remained. An increase in temperature was consequently observed, slightly exceeding room temperature.

### 2.2. SIMS Measurements

The surface film was characterized by Time-of-Flight Secondary Ion Mass Spectrometry (ToF-SIMS). The analyses were performed on the TOF.SIMS 5 instrument (IONTOF, Münster, Germany). Bi^+^ primary ions with an energy of 30 keV were used for the analysis. The current of the Bi^+^ ion beam was between 1.1 and 1.6 pA. The pulse length of the Bi^+^ ion beam was 7 ns, and the mass resolution of the spectrometer (*m/*Δ*m*) was between 5000 and 11,000, depending on the secondary ion species. High mass resolution provided precise identification of secondary-ion signals. Their assignation in the cases of metals and their oxides was further confirmed via the detection of multiple cluster secondary ions. For example, metal oxides form combinations of secondary ions such as MO^−^, MO_2_^−^, MO_3_^−^, M_2_O^−^, M_2_O_2_^−^, M_2_O_3_^−^, M_2_O_4_^−^, etc., where M is a metal. The same pattern can be observed for metal hydrides. The presence of all these secondary ions confirms the composition of the oxide film. Signals originating from organic species were not assigned via fragmentation pathways, since the concentration of specific organic compounds is too low for their individual detection. Instead, the most common fragments connected with organic contaminations were observed, and their assignment was confirmed with their depth profiles.

Depth profiles were measured in a dual-beam depth profiling mode. Cs^+^ ions with the energy of 500 eV were used for sputtering, which allowed us to characterize the composition of ultra-thin passive oxide layers with a thickness of 1–2 nm. The current of Cs^+^ ions was between 33 and 39 nA, resulting in a sputter rate of approximately 0.02 nm/s. The sputter rate was determined with standard samples composed of iron and chromium oxide layers with known thicknesses. Sputtering with the Cs^+^ ions was performed over a 400 µm × 400 µm area, while the analysis with the Bi^+^ ions was performed over a 100 µm × 100 µm area located in the center of the depth profiling crater caused by bombarding with the Cs^+^ ions. Depth profiles were measured in a hydrogen atmosphere with a H_2_ pressure of 7 × 10^−7^ mbar. This approach identifies thin layers of different metal oxides and interfaces between metal oxides and the metallic substrate unambiguously while measuring only negative secondary ions. The initial pressure in the analytical chamber of the SIMS instrument, prior to the H_2_ flooding, was 2 × 10^−10^ mbar, and the purity of the H_2_ was 99.995%. The negative secondary ions were measured during depth profiling.

## 3. Results and Discussion

**Figure 1:**
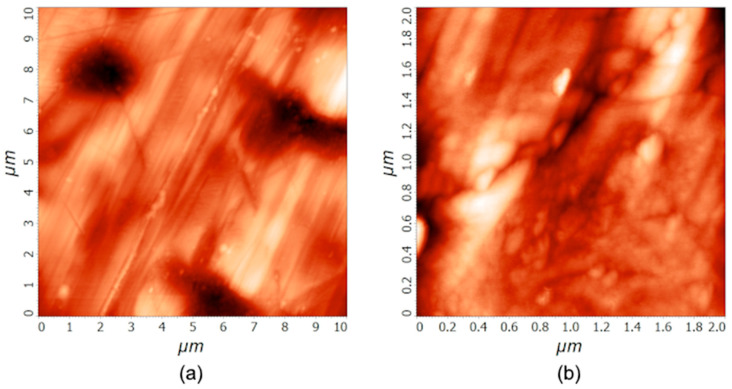
AFM images of as-received stainless steel samples on the surface areas of 10 µm × 10 µm (**a**) and 2 µm × 2 µm (**b**).

Stainless steel samples were characterized by AFM. Figure 1 shows AFM images of an untreated sample at two different scales: 10 µm × 10 µm and 2 µm × 2 µm. Five AFM measurements were conducted for every area size, and the average roughness and standard deviation were calculated. The table, showing all the measured values, is presented in the Supplementary (Table S1). The surface is rather smooth, with some unidirectional scratches. On the smaller scale, the small grains are visible along the scratches. The average roughness *S*_a_, as determined by AFM, was (25 ± 7) nm and (4 ± 1) nm on the areas of 10 µm × 10 µm and 2 µm × 2 µm, respectively.

**Figure 2:**
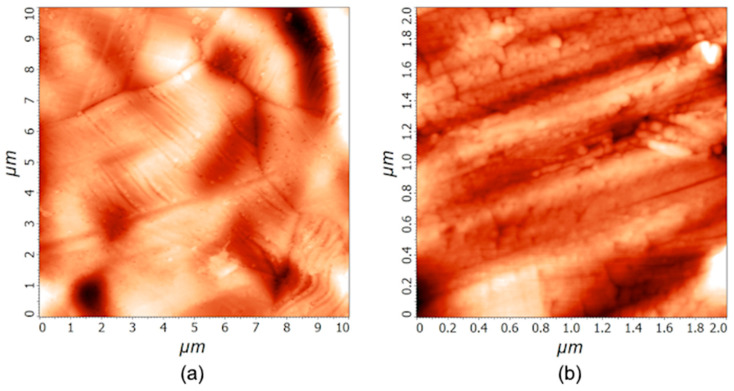
AFM images of stainless steel samples treated in a water solution of 1 M PNA for 60 min on the surface areas of 10 µm × 10 µm (**a**) and 2 µm × 2 µm (**b**).

The treatment with the 1 M PNA solution does not affect the morphological properties of the stainless steel samples significantly, since all the differences are in the range of the statistical error. AFM imaging was performed after the treatment, and the differences between the untreated (Figure 1) and treated samples are small. The average roughness *S*_a_ after 20 min of PNA treatment was (32 ± 15) nm and (4 ± 2) nm for 10 µm × 10 µm and 2 µm × 2 µm areas, respectively. The AFM images acquired at the treatment time of 20 min are shown in the Supplementary (Figure S1), while the images acquired after 60 min of PNA treatment are shown in Figure 2. The surface roughness *S*_a_ after treating the samples for an hour was (34 ± 9) nm and (5 ± 1) nm for 10 µm × 10 µm and 2 µm × 2 µm areas, respectively. The mass of the samples did not change either. We weighed several samples before and after the treatments, and the mass difference was in the range of the experimental error of the analytical balance with a precision of 0.01 mg.

**Figure 3:**
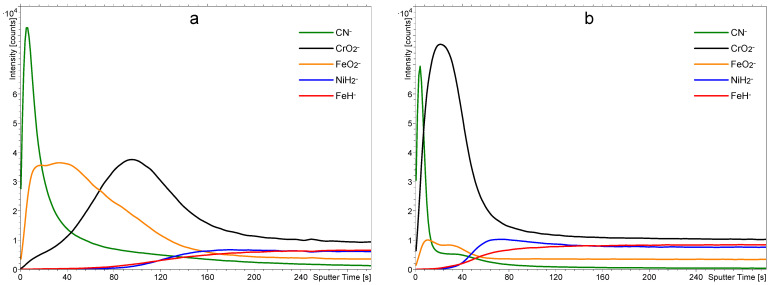
SIMS depth profiles of as-received samples cleaned in a mixture of acetone and isopropanol (**a**) and treated with a 1 M solution of nitric acid for one hour (**b**).

The surface films on stainless steel samples were characterized by ToF-SIMS. A typical depth profile for an as-received sample washed with acetone and isopropanol is shown in Figure 3a, and a profile of a sample treated only with 1 M nitric acid for one hour is shown in Figure 3b. Figure 3a reveals the expected results: There is a film of chromium oxide on the bulk stainless steel, followed by a film of iron oxide. From the sputtering rate of 0.02 nm/s, we estimated the thickness of the Cr-oxide layer to be around 1.4 nm and the same thickness as that of the Fe-oxide (1.4 nm). Together, both oxide layers are approximately 3 nm thick. There is a broad interface between the chromium and iron oxide films, which may be explained either by a mixed layer of chromium/iron oxides or simply as an artifact due to the imperfect smoothness of the samples. As shown in the AFM images in Figure 1, the surface is far from being perfectly smooth, and it is well-known that roughness results in a virtual broadening of interfaces [20,21,22,23]. However, the depth resolution of the Fe_2_O_3_/Cr_2_O_3_ interface is only a few nm, which, considering the SIMS analysis area of 100 µm × 100 µm, is at least an order of magnitude lower than the measured average surface roughness *S*_a_. Ion-sputtering-induced surface roughening should be mentioned as well, since it affects depth resolution. However, an opposite effect of the reduction in surface roughness caused by ion sputtering of non-ideally polished surfaces, such as the stainless steel analyzed, is more probable in this specific situation. This ion-polishing phenomenon, which has an opposite effect on depth resolution compared to roughening, was observed recently on similar types of metallic and metal-oxide samples [20,24].

In the ToF-SIMS spectra, we focused on the signal of CN^−^, which is related to carbon-based contamination on the surface, and the signals of CrO_2_^−^ and FeO_2_^−^, which are related to the Cr-oxide and Fe-oxide present in the passive oxide layers. The signals of NiH^−^ and FeH_2_^−^ are related to Ni and Fe metallic phases present in the bulk of the stainless steel and were formed during the depth profiling because of the hydrogen atmosphere in the analytical chamber. The presence of hydrogen enables effective analysis and differentiation of metals and metal oxides while analyzing only negative secondary ions, since M*_m_*O*_n_*^−^ ions are formed in the layers of oxides while M*_m_*H*_n_*^−^ ions are formed exclusively in the layers of pure metals. Figure 3a shows a film of organic impurities on top of the native oxide film, as revealed by a large signal corresponding to the CN^−^ in the negative SIMS spectrum. There is a rather broad interface between the FeO_2_^−^ and CN^−^ in the depth profile shown in Figure 3a, which is primarily caused by the finite smoothness of the samples.

Figure 3b reveals a SIMS depth profile of a sample treated with 1 M nitric acid at room temperature for an hour. The solution was free from PNA. The depth profile in Figure 3b clearly shows that the nitric acid etched the iron oxide but left the chromium oxide film intact. The result is expected because such a diluted nitric acid does not interact with the chromium oxide, which serves as a corrosion barrier [16]. The organic film in Figure 3b also remains practically intact since the nitric acid does not interact chemically with most organic materials. Therefore, the treatment with a diluted nitric acid at room temperature enables the removal of the iron oxide film but does not affect the film of organic impurities.

**Figure 4:**
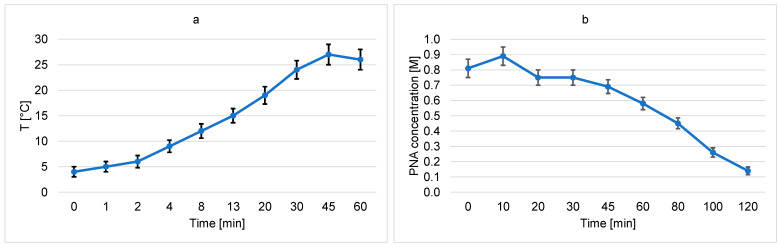
The temperature of the PNA solution versus time (**a**) and the concentration of PNA versus time (**b**).

The samples were treated in a water solution containing approximately 1 M PNA, 1 M NaNO_3_, small amounts of HNO_3_, and residues of H_2_O_2_, which was used during the synthesis in a slight excess in relation to the NaNO_2_. Small amounts of H_2_O_2_ are needed in the reaction mixture to stabilize the PNA. As already mentioned in Section 2, the PNA is unstable at room temperature, so the initial solution temperature when immersing a stainless steel sample was approximately 4 °C. The temperature slowly increased with the increasing treatment time of a stainless steel sample in the PNA solution, and the concentration of PNA gradually decreased by spontaneous decomposition into HNO_3_ and O_2_. Figure 4a shows the temperature of the PNA solution versus time after the immersion of a stainless steel sample. The concentration of PNA in this solution versus the treatment time is shown in Figure 4b.

**Figure 5:**
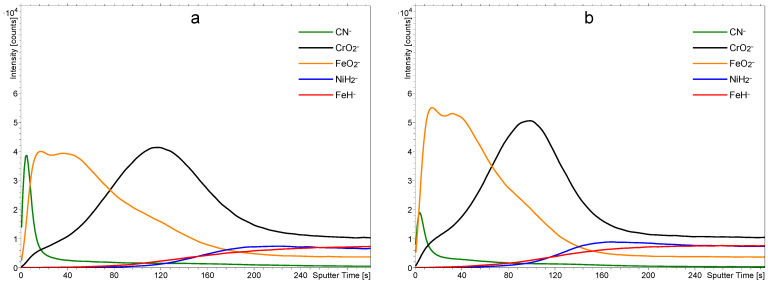
SIMS depth profile of samples treated in a water solution of 1 M PNA for 30 s (**a**) and 240 s (**b**).

Fresh samples of stainless steel were treated with the PNA solutions at different times up to 1 h. A depth profile of a sample treated in a water solution containing 1 M PNA for 30 s is shown in Figure 5a. The intensity of the CN^–^ peak is much smaller than in Figure 3a, so the PNA reacted with the film of organic impurities even at the temperature of 4 °C in such a short time. The removal of the organic impurities is explained by their degradation and oxidation, increasing their polarity and, consequently, solubility in water. As mentioned earlier and supported by the literature on the sterilization of delicate materials with PNA [25], the acid spontaneously releases the HOO radicals, which are one of the reactive oxygen species (ROS). The HOO radicals exhibit a large oxidation potential and interact with organic matter by oxidation [26].

Further degradation of the organic impurities is achieved by longer dipping of stainless steel samples in the 1 M PNA. Figure 5b shows a SIMS depth profile of a sample treated in a water solution containing 1 M PNA for 4 min. The sample temperature was between 4 and 8 °C, as revealed in Figure 4a. The integral intensity of the CN^−^ peak is marginal as compared to the untreated sample (Figure 3a) and also smaller than for the sample treated for half a minute only (Figure 5a). The depth profiles were also determined at other treatment times up to 1 h, and they are presented in the supplementary files in Figures S2–S6. Such a significant change in the intensity of the CN^−^ signal is very likely the consequence of the removal of surface organic contaminations. However, oxidized organic compounds and slightly modified substrates, in combination with the matrix effect, can also influence peak intensities. The most intense effects should be expected from the oxidation since an increase in the oxygen concentration significantly influences the ionization probability of different compounds [27,28]. Nitrated and nitrosylated species formed via PNA action could be the source of nitrogen atoms combining with carbon to form CN^−^ ions. This effect could cause a partial increase in CN^−^ intensity, providing additional argument towards the removal of the overall organic compounds as a requirement for the observed CN^−^ intensity decrease. Much less expressed matrix effect is expected in relation to the metallic substrate, since metal oxide layers are minimally influenced by the PNA treatment. Finally, it should be emphasized that all depth profiles were measured in the H_2_ atmosphere. H_2_ adsorption significantly reduces the initial, substrate-originated matrix effect by the expression of its own matrix effect, which is not sample-specific. The main benefit of the H_2_ matrix is a reliable analysis with more quantifiable results [29], providing additional certainty for depth profiles of PNA-treated stainless steel.

**Figure 6:**
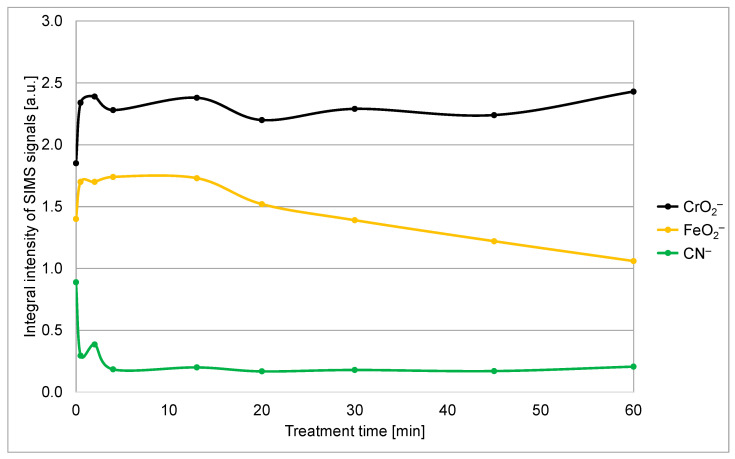
The integral intensities of the CN^−^, FeO_2_^−^, and CrO_2_^−^ signals versus the treatment time in the 1 M PNA solution.

To compare the efficiency of removal of the organic species, we integrated the CN^–^ signal in the SIMS depth profiles over the sputtering time from 0 to 270 s. The integral intensity of the SIMS CN^–^ peak versus the treatment time of stainless steel samples in a solution originally containing 1 M PNA is shown in Figure 6. One can observe a rapid decrease within the first few minutes and stabilization of the integral CN^−^ peak intensity for prolonged treatment time. The finite value of the CN^−^ signal after prolonged treatment might be explained by incomplete oxidation of organic impurities, but a more feasible explanation is secondary contamination since the samples were exposed to ambient air between treatment with the PNA and performing the SIMS depth profiling. Namely, as already mentioned, clean metal (or metal oxide) surfaces are likely to bind organic vapors, which are always present in the ambient atmosphere. Considering this, it is possible to conclude that the treatment of the stainless steel samples with PNA for approximately a few minutes causes almost complete removal of the organic contaminants.

Here, it should be stressed that our samples were pre-cleaned with a rather standard method widely used in laboratories, i.e., treatment in a mixture of pure acetone and isopropanol. Such treatment should remove any greasy or oily dirt that might be on the stainless steel surface. This standard degreasing procedure may be applicable in practice, but the depth profile in Figure 3a shows that traces of organic impurities remain on the surface. These traces are almost completely removed by a brief treatment with 1 M PNA, which could be beneficial in any further treatment where the surface finish, free from organic impurities, is preferred.

Comparison between Figure 3a and Figure 5a,b shows little effect of the treatment in a water solution containing 1 M PNA on the iron oxide film for short treatment times. Namely, the FeO_2_^−^ signal remains practically intact for these treatment times (30 and 240 s). The initial increase in the intensity of FeO_2_^−^ and CrO_2_^−^ signals is an artifact caused by the SIMS measurement and the transient region of depth profiling. The removal of the organic impurities changes the chemical composition of the sample (matrix effect), therefore also changing the ionization probabilities of different secondary ions. Layers closest to the surface are the most affected by this change in the chemical environment.

Figure 6 reveals the integral intensity of this peak versus the treatment time. The measured points are somehow scattered, but the trend is obvious: the signal decreases monotonously with increasing PNA treatment time for treatment times longer than 10 min. Obviously, the treatment causes slow removal of the iron oxide film on top of the chromium oxide film after prolonged treatment. The exposure of Cr_2_O_3_ on the surface of the stainless steel may also improve its corrosion resistance, providing an additional benefit of the PNA treatment. The PNA reacts with the Fe_2_O_3_ at a lower reaction rate than HNO_3_ since it is a weaker acid. Furthermore, the temperature during PNA treatment is lower than during HNO_3_ treatment, additionally decreasing the reaction rate. These are the reasons for the layer of iron oxide to persist on the sample surface even after an hour of dipping in the PNA solution. The integral intensity of the CrO_2_^−^ does not change much for all treatments, which proves that the PNA (at least at the 1 M concentration) is benign to chromium oxide.

The experimental results summarized in this paper show the applicability of the water solution of 1 M PNA for cleaning stainless steel products before any further treatment like coating with various functional films like abrasive-protection, self-lubricant, or corrosion-protective coatings using advanced methods such as physical vapor deposition (PVD by evaporation, sputtering, or ion plating) or chemical vapor deposition (like plasma polymerization). Namely, even a brief treatment lasting only about a minute enables the effective removal of the very thin organic film. The classical degreasing methods using wet chemistry, like non-polar liquids, are time-consuming and ecologically inadequate. Unlike such treatments, the method disclosed in this paper enables ecologically friendly removal of organic impurities since the organic material is oxidized and decomposed.

The brief treatment in a 1 M PNA water solution does not enable the removal of the iron oxide film on top of the chromium oxide, as revealed in Figure 6. Iron oxide is preferentially etched only after prolonged treatment times, which last for an hour or more (when the temperature of the reaction mixture reaches room temperature). Higher temperatures speed up the removal of iron oxide, but PNA is not stable at elevated temperatures, as revealed in Figure 4b. A possible solution useful for the mass application of the innovative method is a brief treatment in the 1 M PNA solution, followed by an immediate treatment with nitric acid or vice versa. The nitric acid could be more concentrated and/or at elevated temperatures, so the removal of the iron oxide film with a water solution of nitric acid could be as fast as the oxidation of the organic impurities by the treatment of stainless steel with the 1 M PNA. A detailed study of such a two-step cleaning process is beyond the scope of this scientific paper since it represents a technological rather than a scientific challenge.

## 4. Conclusions

An ecologically friendly method for cleaning stainless steel was reported. A brief treatment in a water solution of 1 M PNA enables the removal of the surface film of organic impurities, which adsorb on a stainless steel surface upon exposure to the air at ambient conditions. The film is almost completely removed within half a minute of the treatment at about 4 °C. Such a short treatment time does not affect other properties of the samples and does not enable the preferential removal of the topmost iron oxide film while leaving the protective chromium oxide film intact. Prolonged treatment, however, causes slow etching of the iron oxide film on top of the chromium oxide, which may be useful before coating products made from stainless steel with PVD and/or CVD techniques.

## Data Availability

The additional data in the form of the original SIMS spectra and depth profiles, as well as AFM images, will be made available on request. The reason for this is the format in which the measurements are encoded. If the potential users do not have the appropriate software at their disposal, we will personally discuss and decide in which form they want to receive the data (for example, ASCII).

## Supplementary Materials

The following supporting information can be downloaded at: https://www.mdpi.com/article/10.3390/ma18214984/s1, Table S1. Surface roughness (*S*_a_) values measured with the AFM for three different samples over two different areas. Calculated are their average values and standard deviations. Figure S1. AFM images of stainless steel samples treated in a water solution of 1 M PNA for 20 min on the surface areas of 10 µm × 10 µm (a) and 2 µm × 2 µm (b). Figure S2. SIMS depth profile of samples treated in a water solution of 1 M PNA for 2 min. Figure S3. SIMS depth profile of samples treated in a water solution of 1 M PNA for 20 min. Figure S4. SIMS depth profile of samples treated in a water solution of 1 M PNA for 30 min. Figure S5. SIMS depth profile of samples treated in a water solution of 1 M PNA for 45 min. Figure S6. SIMS depth profile of samples treated in a water solution of 1 M PNA for 60 min.
