# Supplementary material for: Rapid Oxidation of Adsorbed Organic Impurities on Stainless Steel by Treatment with Diluted Peroxynitric Acid
Source: Materials (Basel). 2025 Oct 31;18(21):4984. doi: 10.3390/ma18214984 (PMC12609314; doi:10.3390/ma18214984)
Supplement: Supplementary file 1 [file materials-18-04984-s001.zip › materials-3909096-supplementary.pdf]

Supplementary Materials

Table S1. Surface roughness (S_a) values measured with the AFM for three different samples over two different areas. Calculated are their average values and standard deviations.

Place	Ref, 10×10 μm²	Ref, 2×2 μm²	PNA 20 min, 10×10 μm²	PNA 20 min, 2×2 μm²	PNA 60 min, 10×10 μm²	PNA 60 min, 2×2 μm²
1	19.4	4.1	31.0	2.3	46.0	5.0
2	25.0	5.0	57.0	5.6	23.0	3.8
3	20.0	5.1	21.0	4.0	40.0	6.0
4	36.0	2.9	20.7	2.0	28.0	3.0
5	23.0	4.6	31.0	6.5	32.0	5.5
Average	24.7	4.3	32.1	4.1	33.8	4.7
STD	6.7	0.9	14.8	2.0	9.2	1.2

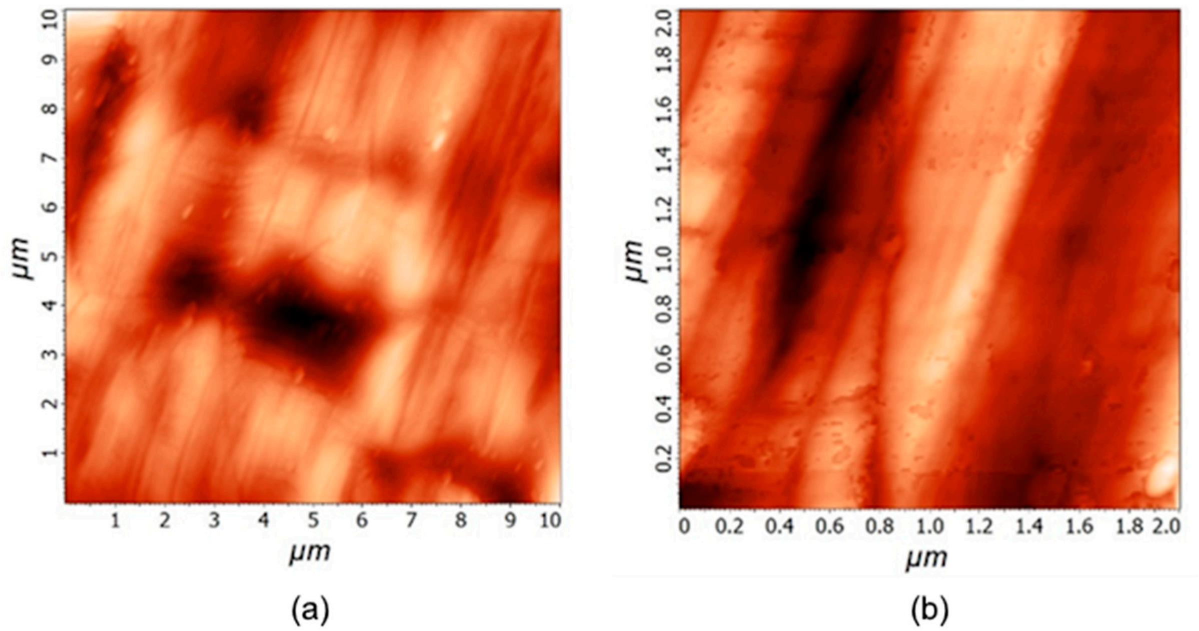

Figure S1. AFM images of stainless-steel samples treated in a water solution of 1 M PNA for 20 min on the surface areas of 10 μm × 10 μm (a) and 2 μm × 2 μm (b).

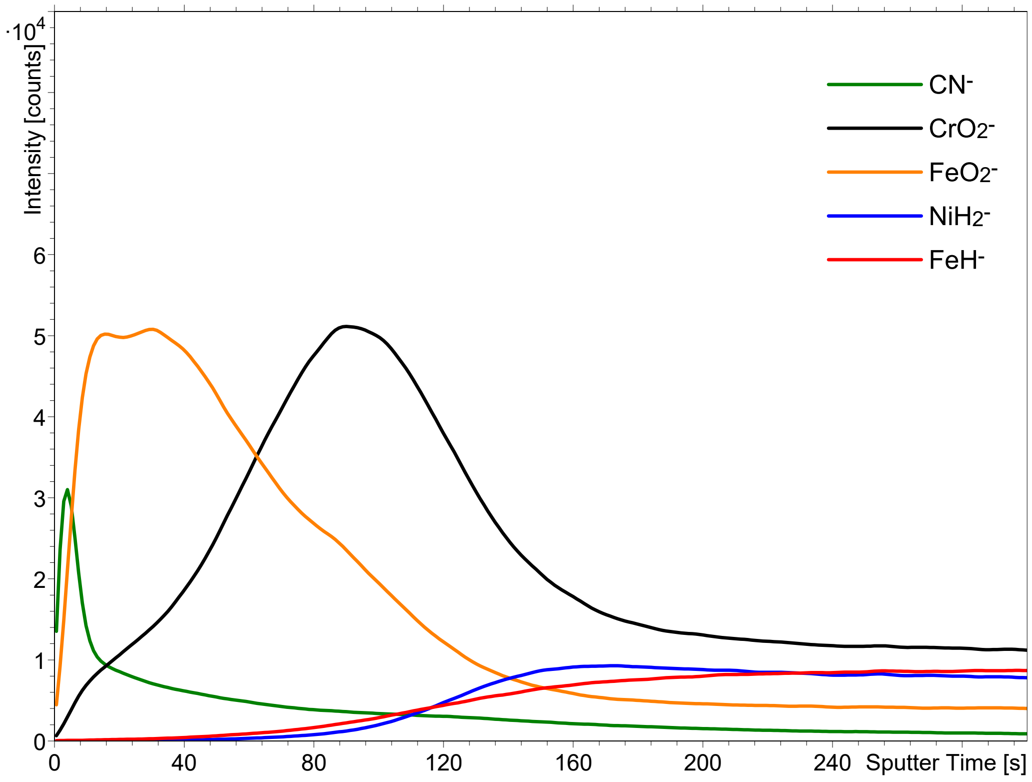

Figure S2. SIMS depth profile of samples treated in a water solution of 1 M PNA for 2 min.

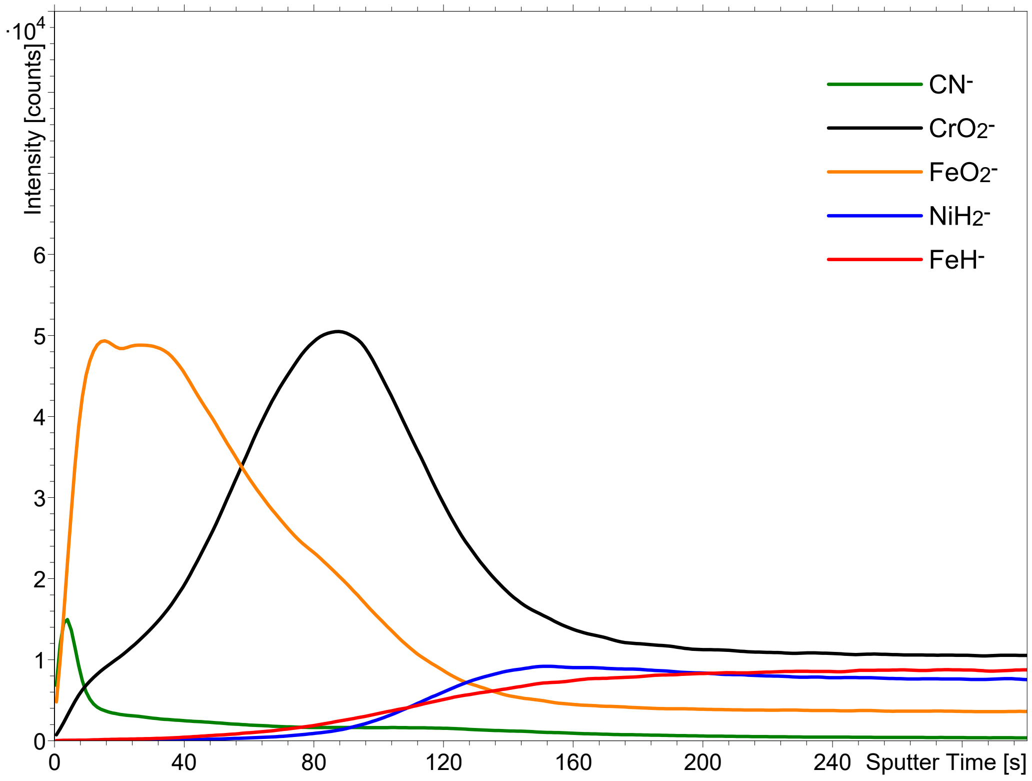

Figure S3. SIMS depth profile of samples treated in a water solution of 1 M PNA for 20 min.

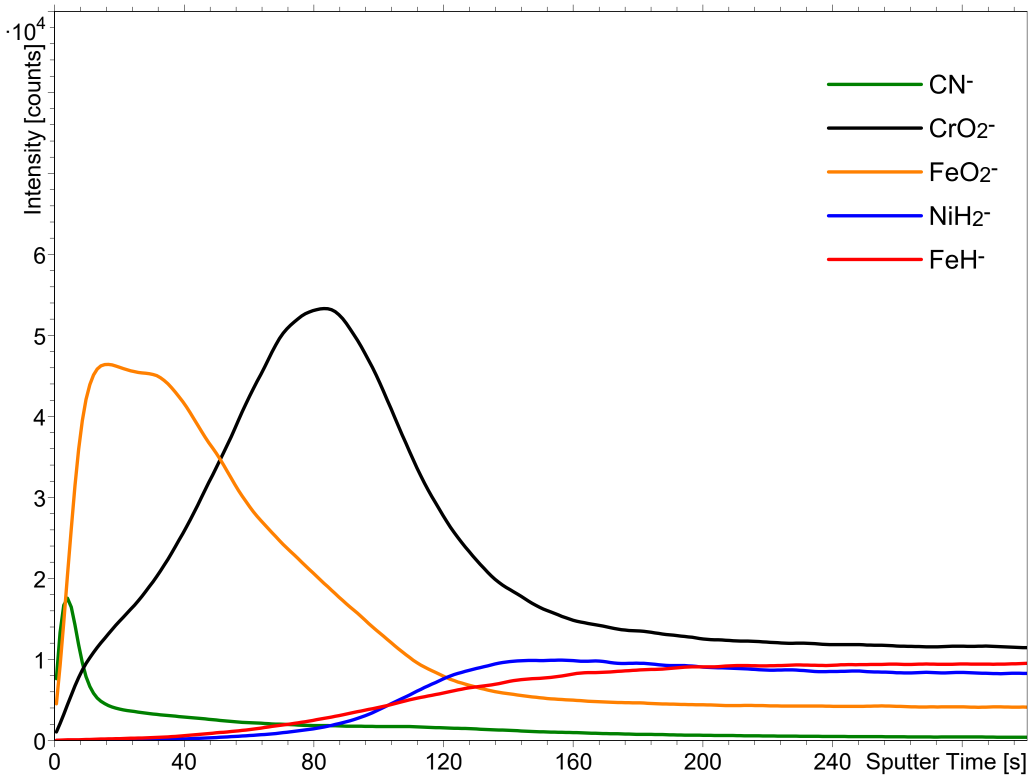

Figure S4. SIMS depth profile of samples treated in a water solution of 1 M PNA for 30 min.

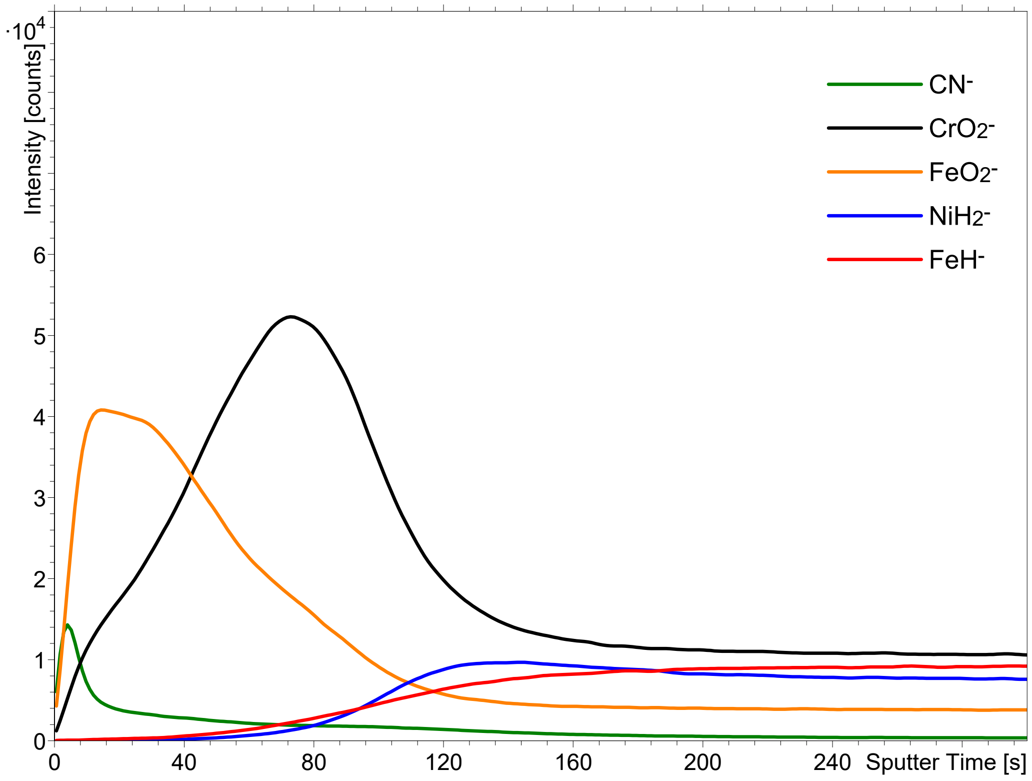

Figure S5. SIMS depth profile of samples treated in a water solution of 1 M PNA for 45 min.

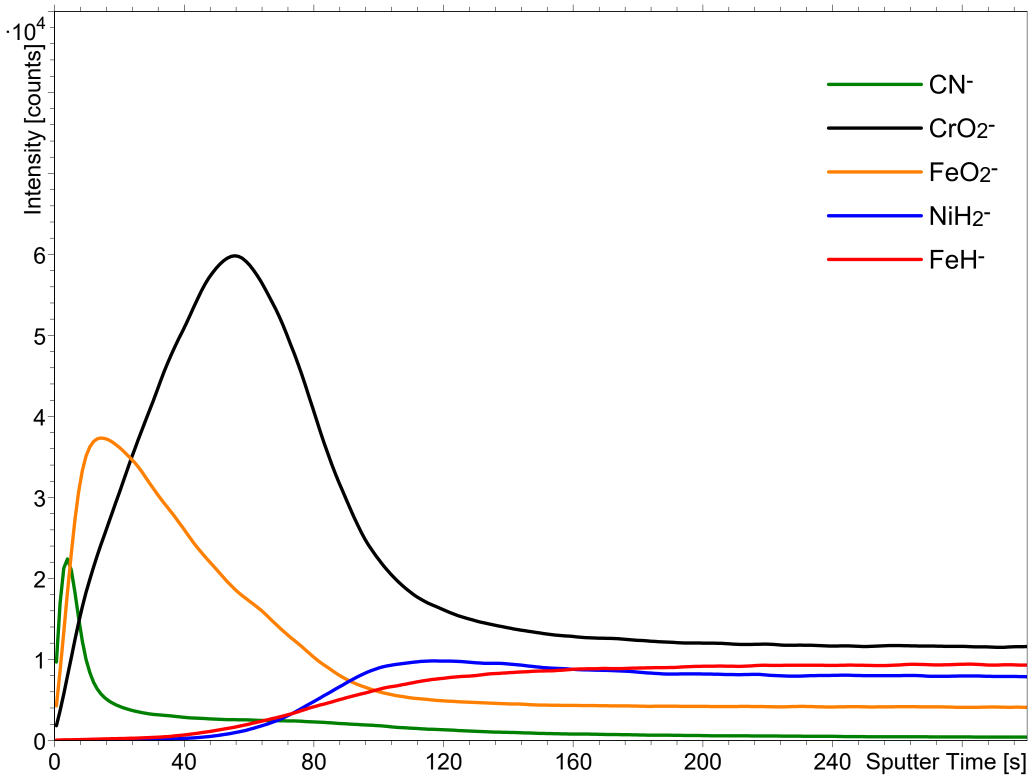

Figure S6. SIMS depth profile of samples treated in a water solution of 1 M PNA for 60 min.
